# Refining Telemedicine: A Plea From Healthcare Workers During a Pandemic

**DOI:** 10.7759/cureus.14664

**Published:** 2021-04-24

**Authors:** Aisha Siraj, Negar Salehi, Saima Karim

**Affiliations:** 1 Cardiovascular Medicine, Case Western Reserve University/MetroHealth Medical Center, Cleveland, USA; 2 Internal Medicine - Department of Cardiology, University of Arkansas, Little Rock, USA

**Keywords:** telemedicine, telephone encounter, video visit, virtual visit

## Abstract

Telemedicine has been in existence for decades with little traction for global mainstream medicine. However, COVID-19 has exposed the importance of providing continued care for patients while minimizing the risk of exposure during pandemics. There has been robust growth in the use of telemedicine since the pandemic began, rendering safety in care provided by minimizing exposure to patients and healthcare workers. There has been tremendous growth and innovation in various digital applications that facilitate telehealth as the platforms continue to improve. Even in the absence of a pandemic, telemedicine allows for care of patients who may live in remote areas or have issues with transportation and comorbidities prohibiting ambulation.

This study is based on a questionnaire for healthcare providers who have been exposed to telemedicine during COVID-19. A survey was shared in social media forums involving groups of physicians and nurse practitioners who were willing to take the survey.

Telemedicine is one of the best approaches to handling situations like pandemics or disease surges. In these circumstances, a virtual visit is beneficial for social distancing if a laptop, smartphone, or tablet is available, along with internet or cellular coverage.

This survey was conducted among healthcare workers of various specialties and it was found out that there was a considerable impetus for the continued benefit from telemedicine as an alternative to in-person visits for selected patients.

Ongoing improvements in the quality of applications/tools, education, and cost are essential to maintain telemedicine. There is also a constant necessity for vast improvements in healthcare policies and reimbursements to allow for telemedicine to evolve.

## Introduction

Despite the existence of telemedicine for years, COVID-19 has accelerated and highlighted the importance of providing continued care for patients while minimizing the risk of exposure during a pandemic. The lack of widespread adoption of telemedicine before COVID-19 is partly because of lack of regulatory framework regarding stepwise initiation, integration into guidelines for practice (especially in conjunction with electronic medical records), funding framework for reimbursement of services rendered, standardized triaging of care, education of public en masse, and safe data sharing mechanism within facilities. While COVID-19 has caused an acceleration in the use of telemedicine tools, there have been pandemics globally prior to COVID-19, and there is a likely possibility of other pandemics after COVID-19 [1-2].

There has been robust growth in the use of telemedicine since the pandemic began, allowing for safe care for patients with minimization of exposure to them, their relatives, and healthcare workers. It has been possible to continue limited in-person appointments due to decreased volume with continued social distancing in patient care areas and waiting rooms. Additionally, telemedicine has reduced the need for personal protective equipment for providers and patients, given the continued national shortage. Telemedicine tools include telephone, video-visits, smartphone data-apps, modules linked with an electronic medical record (EMR), and other less commonly used methods. Examples of applications that integrate into EMR include video visits on Epic, MDlink, American Well, Polycom RealPresence, CyraCom, etc. Examples of applications that do not integrate into EMR are SnapMD, Doximity, Zoom, FaceTime, Skype, WhatsApp, etc. The health insurance portability and accountability action (HIPAA) compliant applications among these include Epic, Doximity, MDLink, Polycom RealPresence.

Even in the absence of a pandemic, telemedicine allows the care of patients who are either unable to get transportation, have comorbidities that prohibit ambulation, or those who live in remote areas without access to providers. Unfortunately, educational and socioeconomic divide has become more prominent between those who can afford or understand how to operate smart devices and have internet or cellular data access. The learning curve of new technology regarding telehealth may also be prohibitive based on prior exposure and age. However, even a telephone encounter may provide adequate ongoing care for a proportion of patients without urgent needs or multiple comorbidities despite the lack of physical exam features that can be detected on video visits. Patient barriers to telemedicine include the desire to see the provider during periods of illness, inability to communicate easily with an established provider, and lack of awareness or access to telemedicine. As far as providers are concerned, the barriers to care include patient education, patient preference for an in-person visit, problems with appropriate triaging, access to technology, and access to approved data applications, challenges with network, reimbursement, decreased productivity, inability to provide physical exam during telephone visits [2].

Triaging and workflow should be tailored to the field of medicine for telehealth to be effective. In places such as urgent care and emergency rooms, “forward triage” allows for effective prospective sorting of urgency before arrival for surge control via screening tools. It also allows for patients with high-risk features of COVID-19 to be isolated immediately upon arrival [3].

On the other hand, patients who are not acutely ill can get their care via telephone or video encounters via various smartphones or apps. Triage is imperative for discerning the need for in-person visit versus virtual visit that occurs in primary care and specialty offices using protocols set in place in most practices depending on diagnosis, the severity of symptoms, urgency of care needed, and number as well as severity of comorbidities [4].

There are two fronts to telemedicine that are acknowledged: asynchronous (with no concurrent communication between provider and patient), and synchronous (live communication between provider and patient). Asynchronous care can include remote monitoring of devices or chronic conditions, exchanging messages via EMR, results of tests being reported/mailed, medication refills, and management. Synchronous care includes in-person, virtual, or telephone visits with a provider. Asynchronous telemedicine has been in place and utilized widely by most practices before the pandemic and continues to be beneficial to patients during the pandemic [5].

Current requirements for telehealth visits include documentation of history, chief complaint, review of systems, past medical history, assessment and plan, level of complexity, mention of any additional workup, total time spent on the encounter (with at least 50% of the time spent in counseling and coordinating care), and examination items (on video visits). Vital signs such a heart rate, blood pressure, respiratory rate, etc., can be added if taken by the patient at home. Adaption of telemedicine within an orthopedic department accelerated by 50% within two weeks, indicates that the systems are user friendly. Standardized checklists to ensure the appropriate use of electronic medical records, cameras/microphones, provision of privacy, templates, patient and provider education, appropriate triaging, and adherence to regulations should be made to allow for the feasibility of rapid implementation [6].

Some factors involved in telemedicine's evolution to make it sustainable, safe and widespread include adequate training of patients/providers/ancillary staff/schedulers/nurses, protocolization triaging, appropriate documentation guides and smart phrases, and data protection confidentiality, safe data sharing practices. Additionally, reimbursement may be a critical factor in the continuity of the provision of telemedicine as well [7]. Even though telehealth has revolutionized medicine, the provider's perspective on its need, user-friendliness, limitations, desire for continuity are missing. We surveyed to involve providers in the discussion to see their view of appropriateness, necessity, and telehealth suitability in their field.

## Materials and methods

This was a cross-sectional study involving a survey for healthcare providers within various fields of medicine. A survey questionnaire (Appendix) was made in SurveyMonkey (San Mateo, CA, USA) (a platform for conducting surveys), and link was shared on facebook (physicians and advanced practice registered nurses [APRNs] in different field of medicine voluntarily answered). Answers from first 200 healthcare providers were accepted who were all in different states of the United State of America. Data was gathered and analyzed. Excel software (Microsoft Corp., Redmond, WA, USA) was used to do statistical analysis.

## Results

This study was based on a questionnaire for providers who have been exposed to the concept of telemedicine during COVID-19. A survey was shared on social media forums involving groups of physicians, nurse practitioners (NPs), and APRNs who were willing to take the survey. In the end, 200 providers from different fields of medicine took the survey.

**Figure 1:**
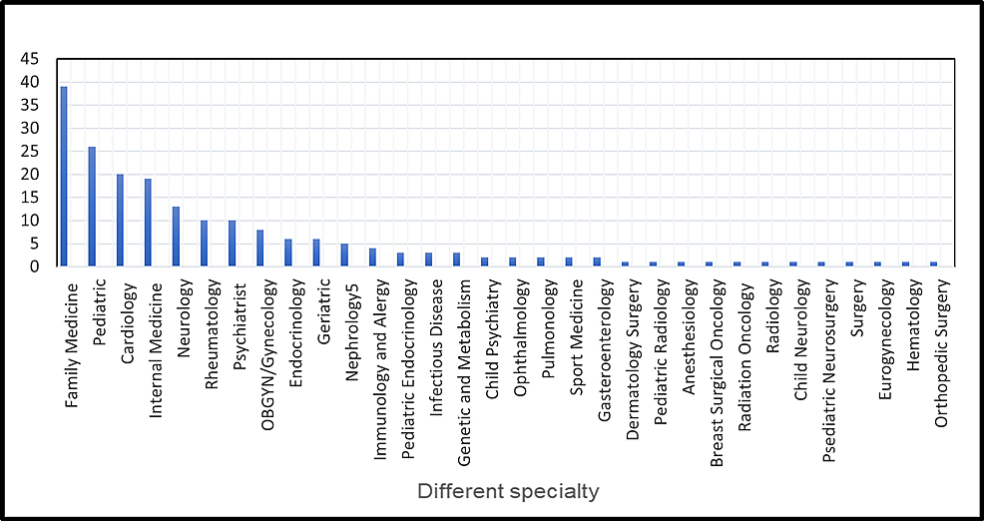
Different specialties in the study

Among those who took the survey, 91.9% were female, 86.5% had an MD degree, and 0.5% of the physicians had a DO degree. There were also 1% APRNs and 1% NPs. 86.5% had completed their training and were practicing independently, while 13.5% were still in training. A myriad of medical specialties was represented in the survey, which is highlighted in Figure 1.

Primary care (including Family Medicine, Internal Medicine, Pediatrics) accounted for 42% of those surveyed. Other larger subsets of specialties represented included Cardiology (10%), Neurology (6.5%), and Psychiatry (5%).

**Figure 2:**
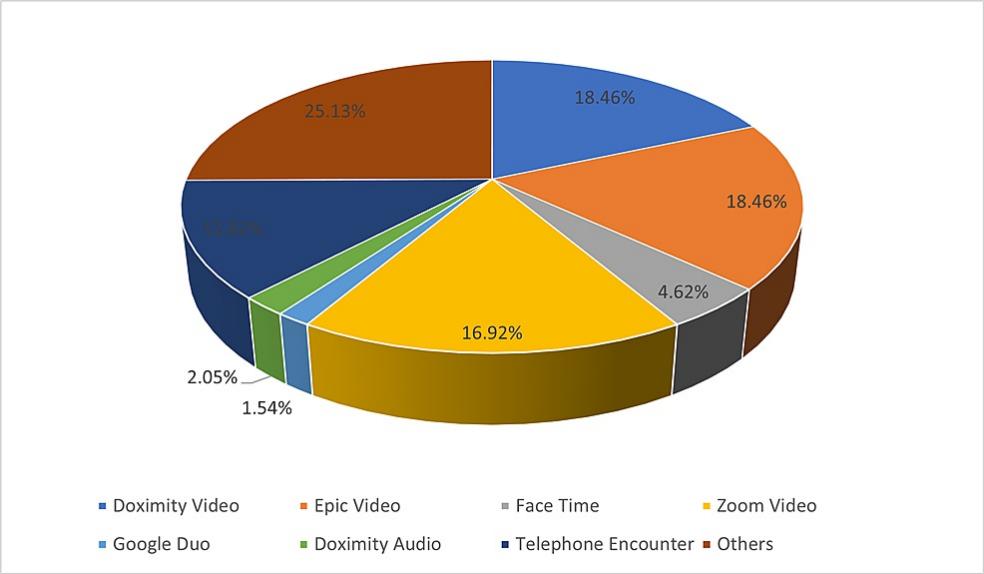
Different applications have been used for telemedicine base on this survey

There were 96.5% of people who took the survey were performing telemedicine. Based on this survey, Doximity and Epic videos are the most common modalities used for telemedicine (18.75% and 18.23%, respectively). However, Zoom video (17.19%), telephone encounter (13.02%) were also common along with Facetime, Google due, and Doximity audio, which was less than 5% each (Figure 2).

71.79% of the medical staff agreed that follow-up patients were the most suitable patients for telemedicine. The second most appropriate telemedicine patients category was individuals with less than three comorbidities (51.79%). 25% of those surveyed believed no difference between a virtual/telephone/app visit or a regular clinic visit. Among those who took the survey, 47.7% thought that doing a physical exam in their specialty is unnecessary, while 42.2% emphasized the importance of physical exams on their visits. The overwhelming majority of health care workers (96.5%) thought telemedicine was a good mode for patient encounters, and most of them agreed that this needs to be part of the training curriculum (90%).

There was an agreement that ancillary staff such as schedulers, triage nurses, clinic nurses, and medical assistants, etc., would continue to provide an invaluable service with telemedicine (81.63%). 93% agreed that telemedicine should be a viable option after the pandemic as well.

**Figure 3:**
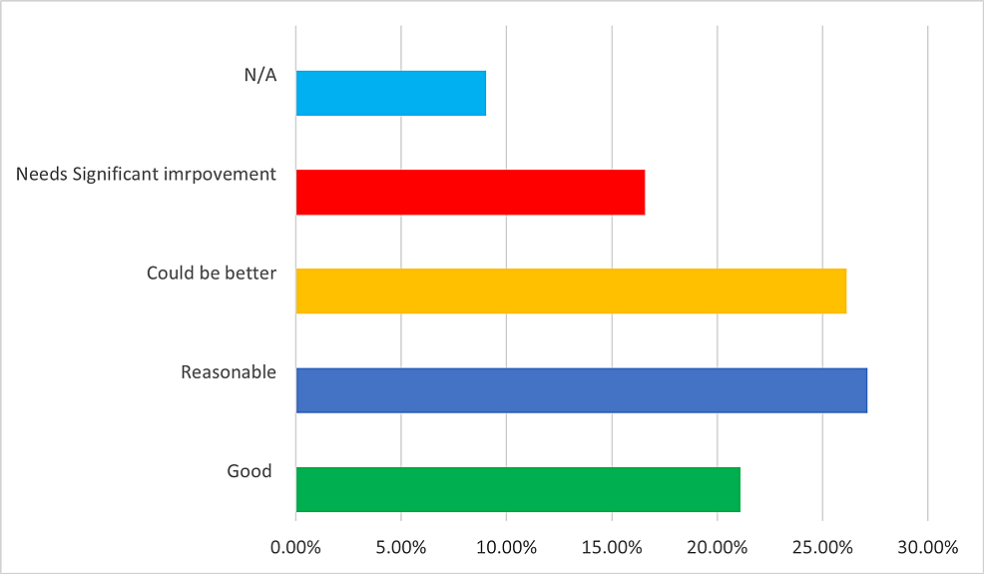
Reimbursement of telemedicine

Telemedicine seemed to have variable effects on providers' productivity. Of all providers, 41% of them agreed that telemedicine increased their work productivity, and 41% mentioned that their efficiency remains the same. At the same time, only about 50% of responders agreed that reimbursement was either good or reasonable, while 26.15% thought it could be better, and 15.9% believed that it needed significant improvement (Figure 3).

As far as research is concerned, 71% thought pandemic had not affected their productivity, while 21.5% agreed that this pandemic decreased their productivity. Interestingly, 60% of survey takers reported that telemedicine helped with improvement of balance of their home and work lives.

## Discussion

Telemedicine is a concept that started in the mid-19th century as communication such as the telegraph and the telephone initiated the possibility of remote medical care. In 1924, when the radio was not widely used and television was not yet invented, an avant-garde illustration of a family communicating with their physician via a video screen was presented in Radio News Magazine [8].

The novel version of telemedicine was created in the mid-20th century from aerospace and military communication technology [9]. At that time, telemedicine was defined by electrocardiogram monitoring, the transmission of radiology images, and remote education. Data transfer was expensive, and lack of appropriate equipment were hindrances until the late 1980-1990s when these barriers were addressed [9].

Telemedicine has been successfully used in several disasters like responses to the Texas hurricane and the Gulf Coast disasters in the fall of 2017. These trials confirmed the availability of access to physicians despite some challenges needed to be overcome. Most of the services, including the prescription of routine medications, evaluation of insect bites, wound infections, and mental health, were managed successfully [10].

In situations such as pandemics or disease surges, telemedicine becomes essential. A virtual visit is beneficial for social distancing if a laptop, smartphone, or tablet is available, along with internet or cellular coverage. Telemedicine utility during COVID-19 is the most recent urgency that has unveiled that virtual visits could be a reasonable replacement for at least 70-80% of follow-up and some of the initial visits.

In this questionnaire, we asked healthcare workers from various specialties with different levels of education about telemedicine. The majority agreed that this could be an excellent tool that should continue after the pandemic with a need to include telemedicine in medical school, graduate, and post-graduate education to instruct regarding different aspects of patient selection, obtainment of appropriate information, the possibility of a physical exam, variety of remote testing/parameters that are feasible, need for escalation of care, etc. One of the limitations of this survey was the absence of data and information from the patients' perspective. Another limitation was that this was a survey that was taken mainly by female healthcare providers. However, the questions asked were not gender-specific and results were likely not affected by the low proportion of male participants, except for questions regarding work-life balance, which innately affects the genders differently in the current healthcare structure.

The survey was instrumental in understanding that healthcare providers appreciate telemedicine and want it as a continued tool after the pandemic. Telemedicine can decrease healthcare costs, increase the level of patients’ satisfaction, provide a better life-work balance for healthcare providers, increase access to care, and provide safe and efficient telephone or virtual visits during pandemics or other dire times. The barriers to telemedicine include potential patient data safety, such as HIPPA compliance, suboptimal algorithms for proper patient selection, poor understanding of remote tools to supplement care (such as vitals done, etc.), and lack of standardized mechanism for escalation of care if needed. Healthcare workers and patients should understand the advantages and disadvantages of telemedicine and traditional care to allow for safe delivery of care.

While everybody is mindful of the requirements for conventional visits, awareness of the tools and setting for telemedicine should also be widespread. HIPPA compliant devices and space that healthcare providers can use for telemedicine are necessary to have an uninterrupted private visit with patients with the provision of ancillary support. Additionally, access and knowledge of tools such as laptops, smartphones, or tablets, HIPPA compliant web-based devices, or applications are necessary for the patient.

Based on this survey conducted for healthcare workers from various specialties, there was a considerable impetus for the continued benefit from telemedicine as an alternative to in-person visits in selected patient groups. Ongoing improvements in the quality of telemedicine forums, supplemental tools, education, and cost are essential to maintain telemedicine's benefits. There is a necessity for vast improvements in healthcare policies and reimbursements that will allow telemedicine to benefit healthcare providers and patients in the future.

## Conclusions

This article highlights the healthcare provider's perspective on telemedicine's benefits and challenges. There is an overwhelming demand amongst physicians and other healthcare providers to continue telemedicine services with involvement of ancillary support staff. COVID-19 has provided us the opportunity to expand access and modes of care that we can provide to our patients. We hope that healthcare workers' plea to expand telemedicine will catalyze further education and improvement in healthcare policies and reimbursement. Telemedicine will likely evolve, endure and expand beyond the pandemic to provide excellent and safe care to larger network of individuals after addressing imminent requirements and overcoming barriers.

## Appendices

General Questionnaire

1-       What is your gender?

- Female

- Male

2-      What is your age:

- <30 years old

- 30-40 years old

- 40-50 years old

- 50-60 years old

- >60 years old

3-      What is your current position?

- Physician MD

- Physician DO

- APRN

- PA

- Nurse

4-      What is your specialty?

5-      Are you current in training?

- Yes

- No

6-      Do you do Tele Health?

- Yes

- No

7-      What modality do you mostly use for Tele Health?

- Doximity Video

- Epic Video

- Facetime

- Zoom Video

- Google Due

- Doximity Audio

- Telephone encounter

8-      What type of patients are more suitable for the Telehealth visit?

-          There is no difference between telehealth visit or regular clinic visit

-          First time visits

-          Follow-up visit

-          Complicated patients (more than 3 core comorbidities)

-          Less complicated cases (less than 3 core comorbidities)

9-      How important is in-person physical exam in your specialty?

- Very important

- Equivalent

- Not Important

10-   With your specialty you think tele health is good option or not?

-          Yes

-          No

11-   Do you think tele-medicine needs to be part of your training curriculum?

- Yes

- No

12-   Do you think Tele Health can be helpful for research purpose between different centers?

-          Yes

-          No

13-   How is reimbursement for telehealth within your healthcare system?

- Good

- Reasonable

- Could be better

- Need Significant improvement

- N/A

14-   Can ancillary staff be incorporated into tele-medicine (Medical assistant, Schedulers, etc...)?

- Yes

- No

15-   Would you like to continue tele-medicine after COVID-19?

- Yes

- No

- Maybe

16-   How has tele-medicine impacted your work productivity?

- Increased

- Decreased

- Same

17-   Has COVID-19 impacted your research activity?

- Increased

- Decreased

- Same

18-   Has tele-medicine changed your salary?

- Increased

- Decreased

- Same

19-   Has tele-medicine during COVID-19 impacted your work life balance?

- Better

- Worse

- Same
